# Extended seasonal prediction of spring precipitation over the Upper Colorado River Basin

**DOI:** 10.1007/s00382-022-06422-x

**Published:** 2022-07-21

**Authors:** Siyu Zhao, Rong Fu, Michael L. Anderson, Sudip Chakraborty, Jonathan H. Jiang, Hui Su, Yu Gu

**Affiliations:** 1 Department of Atmospheric and Oceanic Sciences, University of California, Los Angeles, Los Angeles, CA USA; 2 California Department of Water Resources, Sacramento, CA USA; 3 Jet Propulsion Laboratory, California Institute of Technology, Pasadena, CA USA

**Keywords:** Upper Colorado River Basin precipitation, Sea surface temperature, Extended seasonal prediction, Artificial neural network, Statistical forecast, North American Multi-Model Ensemble

## Abstract

This study provides extended seasonal predictions for the Upper Colorado River Basin (UCRB) precipitation in boreal spring using an artificial neural network (ANN) model and a stepwise linear regression model, respectively. Sea surface temperature (SST) predictors are developed taking advantage of the correlation between the precipitation and SST over three ocean basins. The extratropical North Pacific has a higher correlation with the UCRB spring precipitation than the tropical Pacific and North Atlantic. For the ANN model, the Pearson correlation coefficient between the observed and predicted precipitation exceeds 0.45 (*p*-value < 0.01) for a lead time of 12 months. The mean absolute percentage error (MAPE) is below 20% and the Heidke skill score (HSS) is above 50%. Such long-lead prediction skill is probably due to the UCRB soil moisture bridging the SST and precipitation. The stepwise linear regression model shows similar prediction skills to those of ANN. Both models show prediction skills superior to those of an autoregression model (correlation < 0.10) that represents the baseline prediction skill and those of three of the North American Multi-Model Ensemble (NMME) forecast models. The three NMME models exhibit different skills in predicting the precipitation, with the best skills of the correlation ~ 0.40, MAPE < 25%, and HSS > 40% for lead times less than 8 months. This study highlights the advantage of oceanic climate signals in extended seasonal predictions for the UCRB spring precipitation and supports the improvement of the UCRB streamflow prediction and related water resource decisions.

**Supplementary Information:**

The online version contains supplementary material available at 10.1007/s00382-022-06422-x.

## Introduction

The Upper Colorado River Basin (UCRB), defined as the catchment region upstream of the stream gauge of the Colorado River at Lees Ferry, Arizona, plays an important role in water resources over the southwestern United States (Jacobs 2011). In recent years, a declining trend was observed in the UCRB streamflow during April–July, contributed by sustained drought conditions, in large part due to reduced precipitation (including both rainfall and snowfall) and increase of surface temperature on seasonal scales (Xiao et al. 2018; Hobbins and Barsugli 2020; Milly and Dunne 2020). Currently, the water level at Lake Powell approaches the minimum level (3525 feet) that is required to meet downstream water delivery obligation to the Lower Colorado River Basin under a 100-year-old water-sharing agreement, the Colorado River Compact (Sakas 2021). Consequently, reliable seasonal prediction of the UCRB streamflow becomes increasingly important for water management decisions.

Climate variables such as regional precipitation (rainfall and snowfall) and snowpack have large impacts on streamflow. These variables have been applied to short-lead seasonal predictions of streamflow and water supply for the Colorado River, including those from Natural Resources Conservation Service and Colorado Basin River Forecast Center (e.g., Franz et al. 2003; Pagano et al. 2009; Werner and Yeager 2013; Fleming and Goodbody 2019). Although snow water equivalent in April has the dominant influence on peak flow of the UCRB in April–July, precipitation in spring can significantly influence snow melting and runoff of the UCRB, and so to influence year to year variation of the UCRB streamflow. Thus, a more skillful extended seasonal prediction of precipitation over the UCRB could potentially reduce the uncertainty of its streamflow forecast, especially for the years with strong precipitation anomalies in spring.

To increase the prospect of long-lead forecasts of the UCRB hydroclimate systems (e.g., streamflow and precipitation), previous studies have investigated the relationship between the UCRB hydroclimate systems and large-scale climatic teleconnections, such as those related to sea surface temperature (SST) (e.g., Kim et al. 2006; Regonda et al. 2006; Switanek et al. 2009; Bracken et al. 2010; Kalra and Ahmad 2011; Sagarika et al. 2015, 2016; Zhao et al. 2021). Numerous studies focused on the role of Pacific and Atlantic SST, especially the El Niño–Southern Oscillation (ENSO), Pacific Decadal Oscillation (PDO), and Atlantic Multidecadal Oscillation (AMO) (e.g., Hidalgo and Dracup 2003; Kim et al. 2006; Kalra and Ahmad 2011; Nowak et al. 2012; McGregor 2017; Tamaddun et al. 2017, 2019; Zhao et al. 2021; Zhao and Zhang 2022). An early study showed that the UCRB streamflow has a higher correlation with the AMO compared to other indices associated with the Pacific and Indian Oceans on decadal-to-multidecadal time scales (McCabe et al 2007), while a recent study found a stronger correlation between the Pacific SST and the UCRB streamflow in recent decades on interannual time scales (Zhao et al. 2021). For precipitation, Hidalgo and Dracup (2003) showed that the UCRB precipitation during the warm (cold) season is strongly (weakly) correlated to the El Niño. Similarly, Kim et al. (2006) found that the warm phase of the ENSO is associated with precipitation increase during summer in the UCRB, whereas its cold phase is linked to precipitation reduction during winter in the lower basin. Zhao and Zhang (2022) further showed the causal effect of the tropical Pacific SST in the previous winter on the UCRB spring precipitation using a Granger causality approach. The relationship between the ENSO and Colorado River Basin (CRB) precipitation could be modified by the PDO (Kim et al. 2006).

Taking advantage of oceanic teleconnection, previous studies have used SSTs over multiple basins as predictors to predict the UCRB (or CRB) streamflow and precipitation (e.g., Regonda et al. 2006; Bracken et al. 2010; Lamb et al. 2011; Oubeidillah et al. 2011; Kalra and Ahmad 2011, 2012; Sagarika et al. 2015, 2016; Zhao et al. 2021). For example, Zhao et al. (2021) developed an extended seasonal prediction of the UCRB April–July streamflow with lead times up to nine months by using Pacific SST predictors that have lag-correlation with the streamflow. Their results suggested that the long-lead prediction skill is linked to the strong correlation between the Pacific SST and UCRB streamflow in recent decades. Kalra and Ahmad (2012) adopted a modified version of the Support Vector Machine-based framework to predict annual precipitation (accumulated over 12 months) over the CRB with a lead time of one year using ocean–atmosphere oscillations from 1900 to 2008. Their results showed that using PDO, North Atlantic Oscillation (NAO), and AMO indices as predictors leads to a successful prediction of upper basin annual precipitation, while the AMO and ENSO-related indices can improve prediction skills over the lower basin. Kalra and Ahmad (2011) applied the *k*-nearest neighbor nonparametric technique and found that prediction skills for spring and winter precipitation are better compared to those for the summer and autumn seasons.

The long-lead seasonal prediction of the UCRB precipitation prior to the runoff season (April–July) is critical because water resource decisions often require extended seasonal predictions with a long lead time. For example, the California Department of Water Resources needs predictions of winter and spring precipitation and snow water equivalent in the prior August for its water resource planning. Due to the linkage between SST and UCRB hydroclimate system, the goal of our study is to provide an extended (up to one year) seasonal prediction of the UCRB precipitation using predictors derived from SST over the Pacific and North Atlantic.

Following Zhao et al. (2021), we apply a machine learning tool, i.e., an artificial neural network (ANN), to predict the UCRB precipitation and compare prediction skills of the ANN with those of a stepwise linear regression model, an autoregression model, and three of the North American Multi-Model Ensemble (NMME) models. Specifically, the ANN is able to identify nonlinear relationships among geophysical fields and supplements traditional linear statistical methods in forecasting meteorological and oceanographical fields (e.g., Hsieh and Tang 1998; Hsieh 2001). The stepwise linear regression applies a sequential forward selection approach, which selects predictors in a sequential order by maximizing the total variance explained at each step, and is widely used for seasonal forecasts (e.g., Yim et al. 2015; Li and Wang 2018; Long et al. 2022). Compared to other more advanced linear regression models (e.g., Ridge and Lasso regression), the stepwise regression model does not involve any hyperparameter that needs to be tuned and validated with a large number of samples. The autoregression model and NMME models act as a baseline for the prediction. In addition, the role of soil moisture as a “bridge” between SST and UCRB precipitation will also be examined in this study. As suggested by previous studies, soil moisture in previous seasons may be important to precipitation in the following season (e.g., Beljaars et al. 1996; Zhang et al. 2008; Koster et al. 2016; Yang et al. 2016). Such mechanism of the influence of soil moisture on local precipitation has been evaluated through an atmospheric general circulation model (AGCM) (Koster et al. 2016).

## Data and methods

### Data

The observed monthly averaged precipitation from 1948 to 2019 comes from National Oceanic and Atmospheric Administration (NOAA) Climate Prediction Center (CPC) Unified Gauge-Based Analysis with a spatial resolution of 0.25° (Chen et al. 2008). Note that we aim to predict precipitation during the period of 1980–2019, and the data during 1948–1979 are used for training with retrospective cross-validation. The observed monthly averaged precipitation and soil moisture come from three models (i.e., VIC, Noah, and Mosaic) of Phase 2 of the North American Land Data Assimilation System (NLDAS-2) with a resolution of 0.25° (Xia et al. 2012). The NLDAS-2 dataset was created via the incorporation of observational and reanalysis data into the non-atmosphere coupled land-surface model, and the three-model mean values are used in this study. This study also uses predicted monthly averaged precipitation from three state-of-the-art NMME models, i.e., the CanSIPSv2, COLA-RSMAS-CCSM4, and GFDL-CM2p5-FLOR-B01, with a resolution of 1.0° from 1983 to 2018 (Kirtman et al. 2014). These three models have the longest lead time for predictions of precipitation.

The monthly total natural flow data at Lees Ferry from 1980 to 2018 is obtained from the Bureau of Reclamation (Prairie and Callejo 2005). The monthly averaged SST is obtained from the Hadley Centre Sea Ice and Sea Surface Temperature datasets (HadISST) from 1947 to 2019 at the spatial resolution of 1.0° (Rayner et al. 2003). The monthly mean integrated water vapor transport (IVT) and geopotential height are from 1979 to 2019 from the European Centre for Medium-Range Weather Forecasts fifth-generation reanalysis (ERA5) at the spatial resolution of 2.5° (Copernicus Climate Change Service 2017).

### Hindcast

Our analysis shows that the correlation coefficient between the UCRB April–July streamflow and the UCRB averaged spring precipitation is apparently larger than that between the April–July streamflow and winter precipitation (Fig. S1), indicating that the predictability of the streamflow mainly arises from the UCRB spring precipitation. Thus, we mainly focus on the UCRB spring precipitation in this study. The hindcast of precipitation covers spring (March–May) for the period of 1980–2019. For statistical forecast models, we use observations averaged over February to perform 1-month lead (LD1) predictions and use observations averaged over January to perform 2-month lead (LD2) predictions. Similarly, we use observations averaged over March of the previous year to perform the 12-month lead (LD12) predictions.

For NMME models, real-time forecasts are issued on the 15th of each month using observations on the 1st day of that month (Kirtman et al. 2014). For example, forecasts issued on March 15th, 2019, were initialized by observation on March 1st, 2019. We average the predicted monthly precipitation by NMME models for March, April and May, respectively, as their prediction for spring precipitation. To be comparable with statistical forecasts, the NMME LD1 prediction of spring precipitation is referred to as forecast initialized by March 1st observations issued on the 15th of March. Similarly, the LD2 prediction is referred to as forecast initialized by February 1st observations issued on the 15th of February. Note that the prediction of the statistical forecast model actually has a somewhat longer lead time than that of the NMME model.

### Statistical seasonal predictions

In this study, the seasonal prediction of the UCRB spring precipitation contains three major parts: deriving predictors, creating statistical forecast models, and evaluating prediction skill. The general workflow of this study is similar to that in Zhao et al. (2021), while the predictors and predictand are different. Here the predictors and predictand are SST over three ocean basins (extratropical North Pacific, tropical Pacific, and North Atlantic) and UCRB spring precipitation, respectively. Compared to Zhao et al. (2021) that used Pacific SST as a predictor for the UCRB streamflow, this study includes the North Atlantic SST as an additional predictor to predict the UCRB spring precipitation. The reason we include the North Atlantic SST is that the variability of the North Atlantic SST such as AMO can influence the UCRB hydroclimate system via oceanic-atmospheric oscillation (e.g., McCabe et al 2007; Kalra and Ahmad 2009, 2012).

We first create predictors by calculating the correlation between the UCRB spring precipitation and SST over the three ocean basins. Two cross-validated approaches are applied in this study: leave-three-out and retrospective approaches. For the leave-three-out cross-validation, we leave out three years centered on the year of prediction when calculating the correlation between SST and precipitation. For example, for LD1 of 2000, we use data from 1980 to 1998 and from 2002 to 2019 to calculate their correlation. For years at the beginning or the end, for LD1 of 1980 as an example, we use data from 1983 to 2019 to calculate their correlation. For the retrospective cross-validation, we use data spanning 32 years to calculate their correlation. For example, for LD1 of 1980, we use data from 1948 to 1979 to calculate the correlation. Then, we derive SST predictors for each predicted year at each lead time based on correlation maps between SST and precipitation following Zhao et al. (2021). The steps include: (1) dividing the SST over a selected domain into two groups, one for positive correlations significant at the 95% level and the other for negative, (2) calculating the average SST for each group, and (3) computing the difference between the two groups.

Next, we apply an ANN model and a stepwise linear regression model by using created SST predictors from LD1 to LD12, and an autoregression model by using precipitation from LD1 to LD12, to predict the UCRB spring precipitation. For the ANN, we use a two-layer feed-forward neural network, with a tan-sigmoid transfer function and a linear transfer function in the hidden layer and output layer, respectively. Such selection of the transfer functions is consistent with previous studies (e.g., Tangang et al. 1997, 1998; Hsieh and Tang 1998; Tang et al. 2000; Gaitan et al. 2014). Specifically, 5 neurons are used in the hidden layer and 1 neuron for the output layer. The neural network is trained by the Bayesian Regularization backpropagation algorithm, which minimizes the combination of weights and squared errors. To reduce the effect of nonlinear instability and overfitting, we run the model 10 times and use the 10 ensemble members because the parameters of the neural network are randomly initialized. The stepwise linear regression model sequentially selects predictors by maximizing the total variance of the predictors explained at each step. At the first step, the predictor that is statistically significant at the 0.05 level and has the highest correlation with the predictand is selected. In the next step, we select the predictor yielding the highest correlation coefficient (statistically significant) together with the predictor selected in the previous step. The same procedure is then sequentially repeated until no statistically significant predictor is detected. The autoregression model uses the UCRB precipitation during LD1–LD12 to predict the spring precipitation. Linear regression coefficients are computed between the spring precipitation and leading precipitation and are used for prediction. A detailed description of the three models can be found in Zhao et al. (2021).

We use the two cross-validated approaches to train the ANN and stepwise linear regression models. For the leave-three-out approach, for example, we use SST predictors from February 1983 to February 2019 observations to train the statistical models for the LD1 prediction of spring precipitation in 1980. For the retrospective approach, for example, we use SST predictors from February 1948 to February 1979 observations to train the statistical models for the LD1 prediction of spring precipitation in 1980. In addition, we use the leave-three-out approach to train the autoregression model. Finally, we use three metrics, the Pearson correlation, mean absolute percentage error (MAPE; e.g., Fernández-González et al. 2017; Lv et al. 2019), and Heidke skill score (HSS; e.g., Jury et al. 1999; Yoo et al. 2018), to evaluate prediction skills of forecast models for each predicted year of 1980–2019. The equations of the three metrics are shown below.

1 $${\text{Pearson correlation}} = { }\frac{{{\text{cov}}\left( {{\text{Obs}},{\text{ Hindcast}}} \right)}}{{{\upsigma }_{{{\text{Obs}}}} {\upsigma }_{{{\text{Hindcast}}}} }}{ },$$

where “Hindcast” and “Obs” are predicted and observed precipitation, respectively; ${\text{cov}}\left( { } \right)$ is the covariance between the two variables and ${\upsigma }_{\left( \right)}$ indicates the standard deviation. The MAPE is shown as:

2 $${\text{MAPE }}= \frac{1}{{\text{N}}}\mathop \sum \limits_{i = 1}^{{\text{N}}} \left| {\frac{{{\text{Hindcast}}_{i} - {\text{Obs}}_{i} }}{{{\text{Obs}}_{i} }}} \right| \times 100{\% },$$

where ${\text{N}}$ is total number of years. The HSS is defined as:

3 $${\text{HSS }} = \frac{{{\text{H}} - {\text{E}}}}{{{\text{N}} - {\text{E}}}} \times 100\% ,$$

where ${\text{H}}$ and ${\text{E}}$ are the total and expected number, respectively, of correct predictions of the sign of the normalized precipitation. ${\text{E}}$ sets to be ${\text{N}}/3$ for a random forecast.

## Results

### UCRB precipitation

**Fig. 1:**
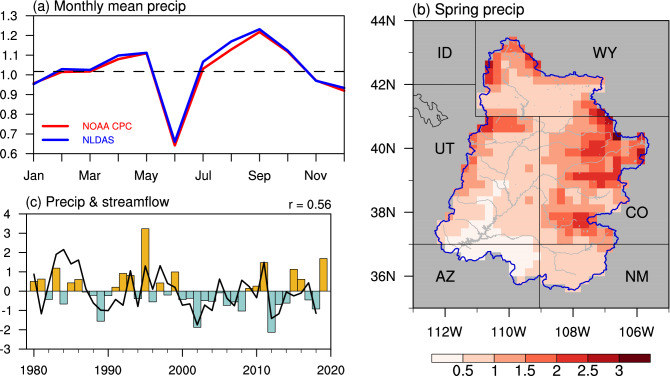
**a** Climatological mean of the monthly mean precipitation (unit: mm day^–1^) averaged over the UCRB using NOAA CPC (red) and NLDAS (blue) datasets, respectively. The black dashed line denotes the climatological mean of annual mean precipitation using NOAA CPC dataset. **b** Climatological mean of precipitation (unit: mm day^–1^) during spring over the UCRB. The abbreviations of the states are labeled. **c** Normalized spring precipitation (bars) averaged over the UCRB and April–July normalized total natural flow at Lees Ferry (black line) for the period of 1980–2019. r represents the correlation coefficient between UCRB averaged spring precipitation and April–July total natural flow

We calculate the climatological mean (for 1980–2019) of both monthly and annual mean domain averaged precipitation (Fig. 1a). The result shows that the monthly mean precipitation has the largest magnitude during spring, late summer, and early autumn, and is not sensitive to the dataset applied. The annual mean precipitation is around 1.1 mm day^–1^ and the spring mean precipitation exceeds this value. It is interesting to note that there is a dip during June. The decrease of rainfall from April to June may be associated with the intensification of the high-pressure system over the western North America, while the increase of the rainfall from June to September is likely due to a northeastward shift of the high-pressure center during the North American summer monsoon (Smith and Kummerow 2013). The similarity between UCRB precipitation (especially in spring) in NOAA CPC dataset and NLDAS dataset indicates that the result is not sensitive to dataset applied. In the following study, we will focus on the spring season and use the NOAA CPC dataset.

Figure 1b shows the long-term climatology of the spring precipitation over the UCRB. The precipitation with large values (> 1.5 mm day^–1^) appears over boundaries of the UCRB and provides water resources for the Colorado River and its branches. The correlation between the UCRB averaged spring precipitation and April–July natural flow at Lees Ferry is also calculated, with a correlation coefficient of 0.56 (Fig. 1c). Given its significant correlation with the UCRB April–July streamflow (Hoerling et al. 2019), we will focus on the UCRB averaged spring precipitation in the following analyses.

### Role of SST in the UCRB spring precipitation

**Fig. 2:**
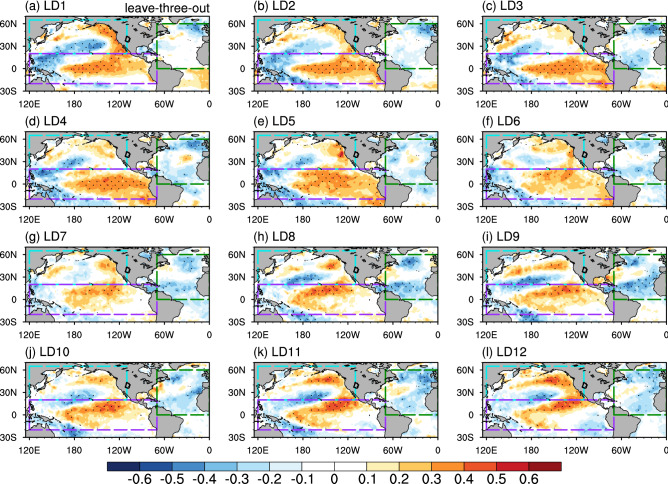
**a** The correlation coefficient (shading) between SST and UCRB spring precipitation at LD1 using the leave-three-out cross-validation averaged for the period of 1980–2019. Black dots represent correlation coefficients significant at the 95% level. The blue, purple, and green boxes represent the extratropical North Pacific, tropical Pacific, and North Atlantic, respectively. **b–l** Same as in **a**, but from LD2 to LD12

Following previous studies that highlight the effect of the Pacific and North Atlantic SSTs (especially those related to ENSO, PDO, and AMO) on the UCRB precipitation (e.g., Hidalgo and Dracup 2003; Kim et al. 2006; Kalra and Ahmad 2011; Nowak et al. 2012; McGregor 2017; Tamaddun et al. 2017, 2019; Zhao et al. 2021; Zhao and Zhang 2022), we calculate the correlation coefficient between the UCRB averaged spring precipitation and SST for LD1–LD12. Figure 2 shows the correlation coefficient averaged over the period of 1980–2019 using the leave-three-out cross-validation. In general, the correlation coefficient over the Pacific exhibits a tri-pole pattern, with positive correlations over the coast of North America and the tropical Pacific, and negative correlation over around 30°N, while the correlation coefficient over the North Atlantic is generally negative. To further examine the robustness of the SST patterns, we calculate the correlation coefficient between SST and precipitation using the retrospective cross-validation. The averaged correlation map shows similar patterns, despite smaller magnitude of the correlation (Fig. S2).

We create SST predictors for the extratropical North Pacific (20° N–65° N, 120° E–110° W), tropical Pacific (20° S–20° N, 120° E–70° W), and North Atlantic (0°–60° N, 70° W–0°), respectively. We select the three basins to create SST predictors because (1) the correlation coefficients over the three basins are relatively high and (2) the linkage between SST over the three basins and UCRB precipitation is well documented (e.g., Hidalgo and Dracup 2003; Kim et al. 2006; Kalra and Ahmad 2011, 2012; Nowak et al. 2012; McGregor 2017; Tamaddun et al. 2017, 2019). The change of SST predictors is very small when we alter the boundary of the SST domain by several degrees. As described in the methodology (Sect. 2.3), the SST predictor varies each year and lead time.

**Fig. 3:**
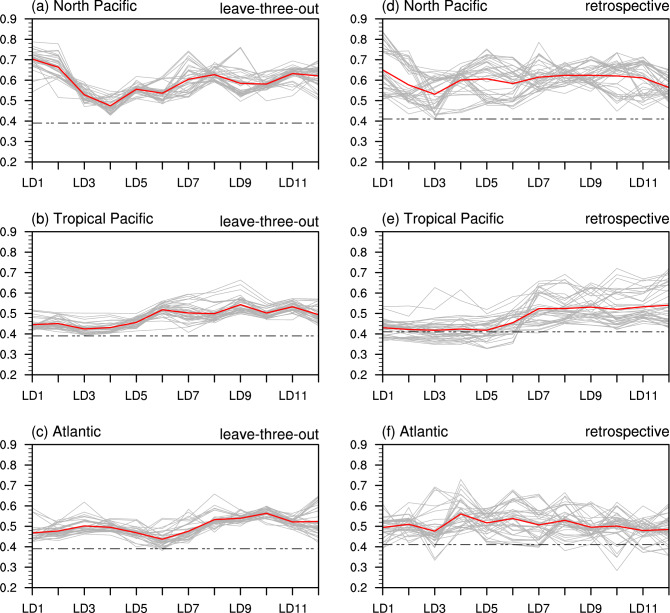
**a** Correlation coefficients between the North Pacific SST predictors and UCRB averaged spring precipitation from LD1 to LD12 for 1980–2019. The gray lines denote correlations between the two variables using the leave-three-out cross-validation. There are a total of 40 gray lines and each line represents one year during 1980–2019. The red line represents the average of all the 40 years. **b** and **c**, Same as in **a**, but for the tropical Pacific and Atlantic SST predictors. **d–f**, Same as in **a–c**, but for the retrospective approach. The black dashed lines represent correlation coefficients significant at the 99% level

Figure 3a–c shows the correlation coefficients between SST predictors for each of the three ocean basins and the UCRB averaged spring precipitation for each predicted year at each lead time using the leave-three-out approach. Average correlation coefficients (red line) are all greater than 0.40 and significant at the 99% level. In general, the average correlation coefficient of the extratropical Pacific SST predictors is higher than that of the other two ocean-basin predictors, indicating that the extratropical Pacific plays a more significant role in the UCRB spring precipitation than do the tropical Pacific and North Atlantic. The average correlation coefficient of the extratropical Pacific SST predictors drops quickly from 0.70 at LD1 to 0.47 at LD4 but increases somewhat to around 0.50–0.60 afterward, consistent with the evolution of correlation patterns for the extratropical Pacific (Fig. 2). In addition, we also calculate correlation coefficients between the UCRB averaged spring precipitation and SST predictors for the three ocean basins together (Fig. S3a) and global oceans (Fig. S3b), respectively, for each predicted year at each lead time using the leave-three-out approach. The correlation coefficients are much smaller than those for extratropical Pacific SST predictors because the large contribution of the extratropical Pacific is unable to stand out when it is mixed with other ocean basins.

Figure 3d–f shows correlation coefficients between SST predictors for each of the three ocean basins and the UCRB averaged spring precipitation for each predicted year at each lead time using the retrospective approach. The retrospective approach shows a larger spread of each year’s correlation coefficient (gray line) than that of the leave-three-out approach, but their average correlation coefficients (red line) are similar. Such a larger spread of the correlation is due to longer data records used in the retrospective approach. This result suggests that the decadal variability of the correlation between the UCRB spring precipitation and SST contributes to the larger spread among different years, as discussed in Zhao et al. (2021). In addition, we also compute the correlation between the UCRB average spring precipitation and other climatic indices over Pacific and Atlantic for LD1–LD12. These indices include PDO, Oceanic Niño Index (ONI), AMO, North Pacific Index (NPI), North Pacific Gyre Oscillation (NPGO; similar to the second empirical orthogonal function mode of North Pacific SST––the Victoria Mode), and Atlantic Niño Index (ATL3). The result shows that their correlations range from − 0.50 to 0.40 (Fig. S4), significantly lower than those of SST predictors (especially the North Pacific SST predictors) derived in this study (Fig. 3).

It is noted that there is a dip in the correlation coefficient in Fig. 3 for short lead times (e.g., LD4–LD6) but a higher correlation at longer lead times (e.g., LD10–LD11). Following previous studies that suggested that soil moisture in previous seasons may be important to precipitation in the following season (e.g., Beljaars et al. 1996; Zhang et al. 2008; Koster et al. 2016; Yang et al. 2016), here we hypothesize that the long memory of soil moisture is responsible for the higher correlation between SST and UCRB spring precipitation for the long lead times.

**Fig. 4:**
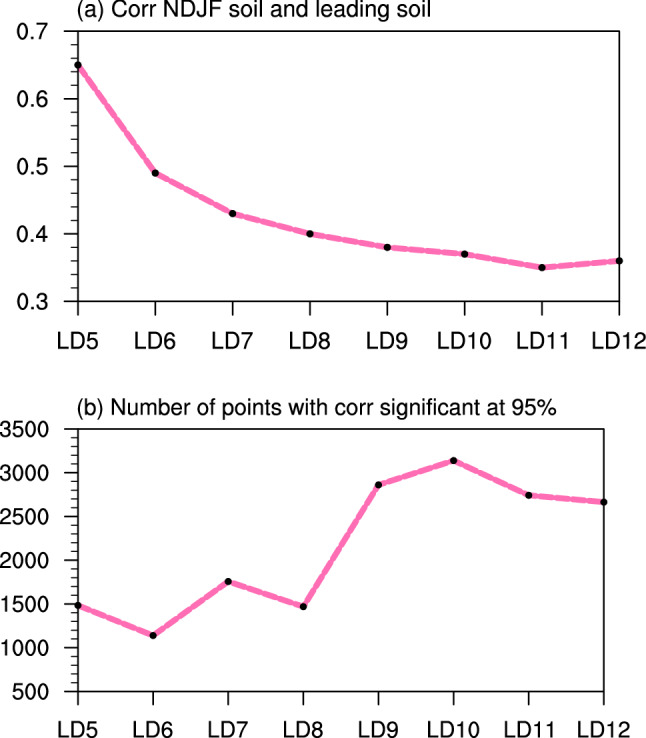
**a** Correlation coefficients between UCRB averaged soil moisture in NDJF and that in LD5–LD12. **b** The number of points over the domain of the three ocean basins with correlation coefficients (between the UCRB averaged soil moisture and SST over the three basins at the same lead time) significant at the 95% level. In other words, we first calculate the correlation coefficient between the UCRB averaged soil moisture at one lead time (e.g., LD5) and SST over the three basins at that lead time (e.g., LD5). Then, we calculate the total number of grid points with correlation coefficient significant at the 95% level

To examine this hypothesis, we first calculate the correlation between UCRB averaged spring precipitation and UCRB averaged November–February (NDJF) soil moisture for the period of 1980–2019. Their correlation is 0.35 (significant at the 95% level), indicating that the NDJF soil moisture is significantly correlated to spring precipitation over the UCRB. The mechanism of the influence of soil moisture on local precipitation has been investigated via an AGCM (Koster et al. 2016). Next, we calculate the correlation between UCRB averaged soil moisture in NDJF and that in the leading times (LD5–LD12; note that these lead times correspond to the spring precipitation) to investigate whether soil moisture has a long memory (i.e., autocorrelation). Their correlation coefficients range from 0.65 (for LD5) to 0.35 (for LD10–LD12) (significant at the 95% level) (Fig. 4a), indicating that the soil moisture’s signal at LD10–LD12 (i.e., previous spring) can persist through the following winter (i.e., NDJF). Then, we compute the correlation between the UCRB averaged soil moisture and SST over the three basins for each lead time of LD5–LD12 (these lead times correspond to the spring precipitation). The result shows that the correlation between soil moisture and SST at short lead times (e.g., LD5–LD6) is generally lower than that at long lead times (e.g., LD9–LD12) (Fig. S5). Such a result is confirmed when we count the number of grid points over the three ocean basins with correlation coefficients significant at the 95% level: the number for long lead times is much larger than that for short lead times (Fig. 4b).

The above result may be counterintuitive, and is presumably due to seasonal variation of the teleconnection. SST in previous September–October (LD5–LD6) generally has a weaker remote influence on the UCRB soil moisture (at LD5–LD6) and thus spring precipitation than SST in March–June (LD9–LD12) does. The long memory of soil moisture (Fig. 4a) appears to preserve the SST influence from the previous spring/early summer to winter (NDJF) and then influence the precipitation in the following spring. Thus, the high correlation between SST and UCRB spring precipitation for long lead times (Fig. 3) is probably due to (1) high correlation between SST and UCRB soil moisture at these lead times and (2) such SST–soil moisture information persisting into the following spring (Fig. 4).

**Fig. 5:**
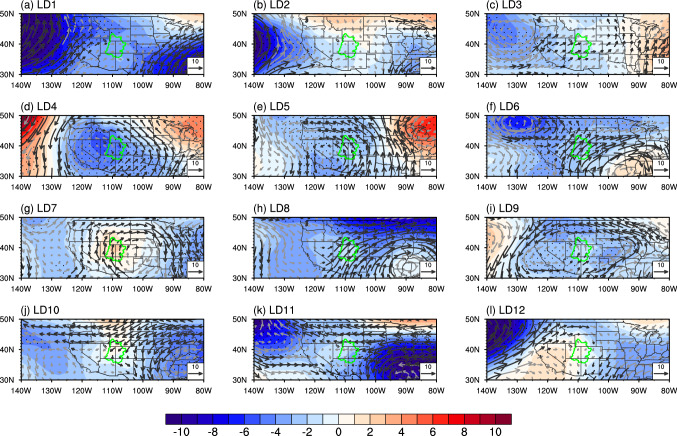
**a** Regression of IVT (vectors; unit: kg m^–1^ s^–1^) and geopotential height at 850 hPa (shading; unit: m) at LD1 onto the UCRB averaged soil moisture at LD1. For IVT, values significant at the 95% confidence level or higher are shown in black vectors, where gray vectors indicate values not significant. For geopotential height, stippling indicates regions significant at the 95% level. The green curve indicates the boundary of the UCRB. **b–l** Same as in **a**, but for the regression of IVT and 850 hPa geopotential height onto the UCRB averaged soil moisture with the same lead time for LD2–LD12, respectively

We further examine how IVT is linked to soil moisture by regressing the IVT for each lead time of LD1–LD12 onto the UCRB averaged soil moisture for each lead time of LD1–LD12 (Fig. 5). For most of the lead times (e.g., LD1–LD9 and LD12), the IVT originated from the south, west or southwest is linked to soil moisture over the UCRB, while the IVT from the east is associated with the soil moisture for LD10–LD11. To understand the physical mechanism associated with the IVT–soil moisture linkage, we further investigate the regressed geopotential height at 850 hPa. In general, the UCRB soil moisture is accompanied by low pressure anomalies in the lower troposphere (850 hPa) over the eastern Pacific or the United States. Such low pressure anomalies may induce IVT from the south, west or southwest (e.g., LD1–LD9 and LD12). For LD10–LD11, the IVT originated from the east is accompanied by westward extended low pressure anomalies located over the eastern United States (Fig. 5j–k). Due to the long memory of soil moisture (Fig. 4a), those atmospheric features (IVT and geopotential height) in long lead times can potentially be linked to soil moisture in winter (i.e., NDJF) and thus spring precipitation.

### Prediction skills of precipitation

We perform extended seasonal predictions for the UCRB spring precipitation using normalized SST predictors of the three ocean basins. The discussion in previous section suggests that soil moisture can act as a “bridge” between SST and UCRB precipitation. The correlation between the UCRB averaged soil moisture from LD1 to LD12 and UCRB averaged spring precipitation is less than 0.40. Thus, we will only use SST predictors of the three ocean basins as predictors. We compare the prediction skills of the ANN model with those of the stepwise linear regression model, the autoregression model, and three NMME models. Three verification metrics are used to evaluate prediction skills.

**Fig. 6:**
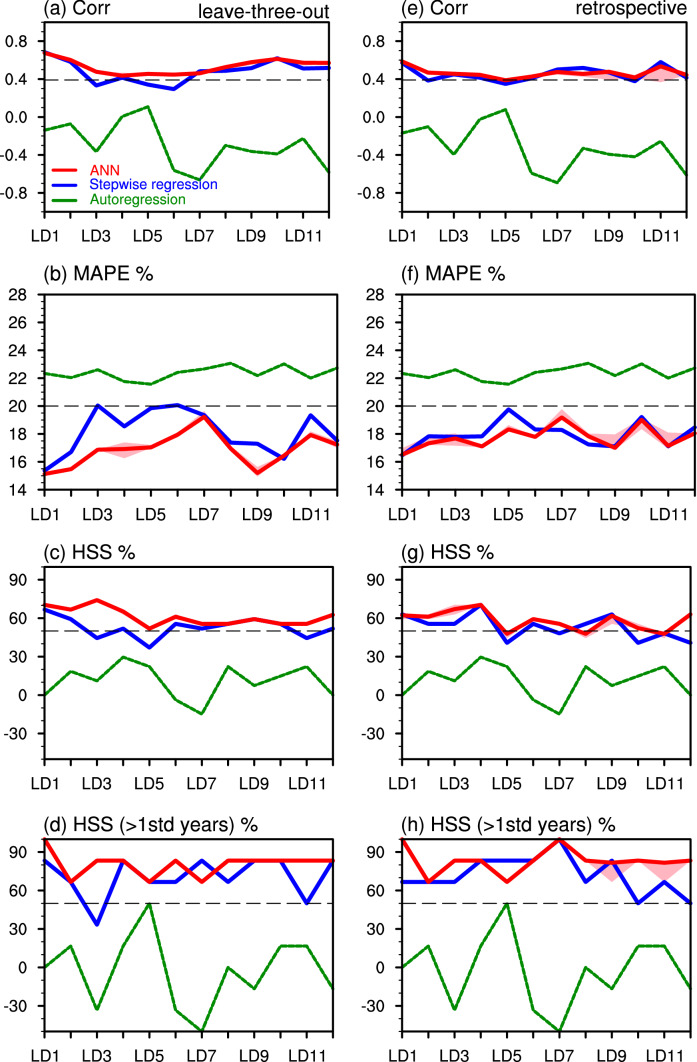
**a** The correlation coefficients between the observed and predicted precipitation from LD1 to LD12 using the leave-three-out cross-validation. The red and blue lines denote results from the ANN (ensemble mean) and stepwise linear regression, respectively, by using SST predictors of the three ocean basins. Shading denotes the spread of the maximum and minimum values of 10 ensembles of the ANN. The green line represents the result from the autoregression. **b–d** Same as in **a**, but for MAPE (%), HSS (%) for all years, and HSS (%) for anomalous years, respectively. **e–h** Same as in **a–d**, but for the retrospective cross-validation. The black dashed line represents a satisfactory prediction, that is, Pearson correlation > 0.40, MAPE < 20%, and HSS > 50%

Figure 6a exhibits correlation coefficients between the observed and predicted precipitation from LD1 to LD12 using the leave-three-out cross-validation. For the ANN model (with 10 ensemble members), Pearson correlation is above 0.40 (*p*-value < 0.01) for LD1–LD12, with high correlations (> 0.50) for LD1–LD2 and LD8–LD12, consistent with the higher correlation between the extratropical Pacific SST predictors and precipitation for these lead times (Fig. 3a). This result confirms the dominant role of the extratropical North Pacific in the UCRB spring precipitation. The correlation coefficient of the ANN is generally higher than that of the stepwise linear regression model, especially for LD3, LD5, and LD6. The autoregression model has no skill (correlation < 0.10). The MAPE of the ANN is less than 20%, indicating that the magnitude of precipitation is well captured, while the MAPE values in the other two approaches are larger (Fig. 6b) than that of ANN. In addition, we also use six oceanic indices (PDO, ONI, AMO, NPI, NPGO, and ATL3) from LD1 to LD12 as predictors to predict the UCRB spring precipitation by applying the ANN. The result is listed in Table S1 and the skill is lower than that using SST predictors of the three ocean basins as predictors.

For the retrospective approach, the Pearson correlation is slightly smaller and MAPE is slightly larger than those of the leave-three-out approach (Fig. 6e, f). The lower skills for the retrospective approach may result from the larger spread of the correlation in this approach (see Fig. 3). Additionally, both the correlation and MAPE are similar between the ANN and stepwise linear regression model for the retrospective approach. The HSS for all years and anomalous years (precipitation anomalies outside the ± 1 standard deviation) ranges from 40% to 80% and 50% to 100%, respectively, for LD1–LD12, generally greater than 50% that is the threshold for a satisfactory prediction (Jury et al. 1999; Zhao et al. 2021) (Fig. 6c, d, g, h). The HSS of the ANN is slightly higher than that of the stepwise linear regression model for both cross-validated approaches.

**Fig. 7:**
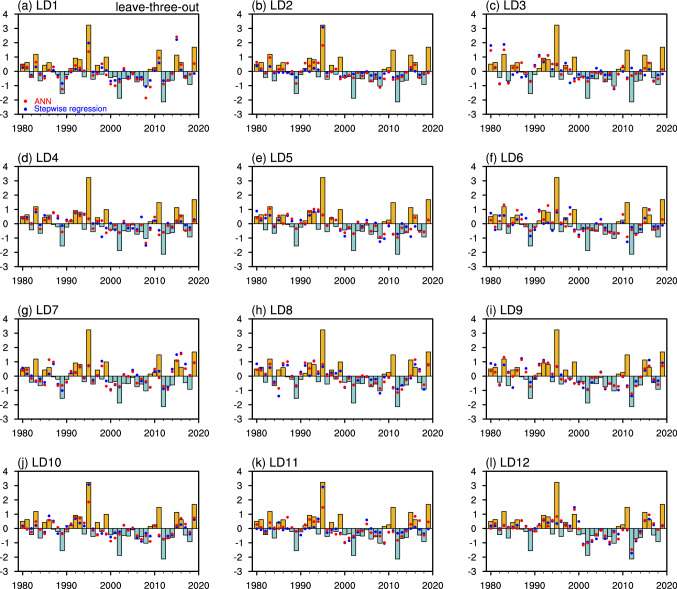
**a** The observed normalized UCRB spring precipitation (bars) and predicted results by the ANN (red dots) and stepwise linear regression (blue dots) by using SST predictors of the three ocean basins at LD1 from 1980 to 2019 with the leave-three-out cross-validation. **b–l** Same as in **a**, but from LD2 to LD12

Figure 7 shows the hindcast of normalized precipitation anomalies for LD1–LD12 for individual years compared to those observed using the leave-three-out cross-validation. In general, both ANN and stepwise linear regression models can capture the sign of the observed precipitation anomalies, giving rise to the prediction skills shown in Fig. 6. Consistent with the HSS, the sign of normalized precipitation is better predicted by the ANN than the stepwise linear regression model for LD3, LD5, and LD6. Moreover, the sign of precipitation anomalies for extreme years is well captured (e.g., 1989, 1995, 2002, 2012, and 2019). The statistical models also well capture the magnitude of precipitation anomalies for neutral years. However, precipitation anomalies with very large magnitudes are generally underestimated. For example, the hindcasts only predict about a half or less of the positive precipitation anomalies in 1995, the largest magnitude during the 40 years, with only the exception at LD2, LD10, LD11 for the linear model. Similarly, the hindcasts underestimate the large negative precipitation anomalies in 2002 and 2012 and the positive anomalies in 2011 and 2019. Similar results could be seen for predicted precipitation anomalies for individual years using the retrospective approach (Fig. S6).

**Fig. 8:**
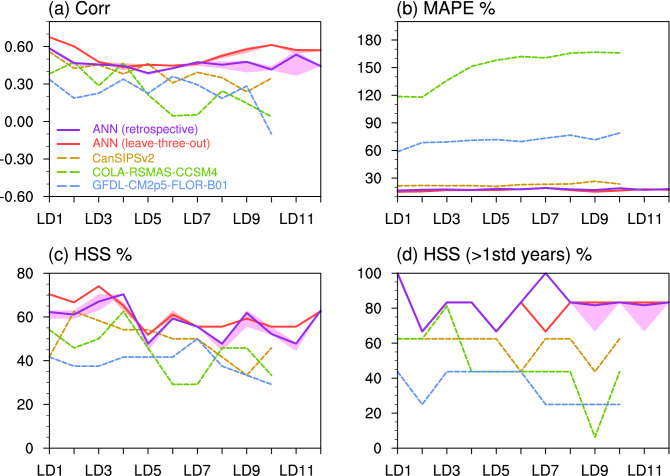
**a–d** Same as in Fig. 6a–d, but for verification metrics from the ANN with the retrospective approach (purple), ANN with the leave-three-out approach (red), CanSIPSv2 (brown), COLA-RSMAS-CCSM4 (green), and GFDL-CM2p5-FLOR-B01 (blue). Only LD1–LD10 is available for the three NMME models

Finally, we compare verification metrics of the ANN model (with the two cross-validated approaches) with those of the three NMME models, which provide forecasts of spring precipitation with lead times up to 10 months. All the three NMME models show decreasing Pearson correlations from LD1 to LD10 (Fig. 8a), consistent with typical prediction skills of dynamic models when lead time is longer (e.g., Zhao and Yang 2014, Zhao et al. 2015). Such low skills at long lead times may be associated with (1) initial conditions for these lead times (as initial conditions are responsible for the initial bias growth; Ma et al. 2021) and (2) model drifts (e.g., asymptotic, overshooting, and inverse drift; Hermanson et al. 2018; Manzanas 2020). The CanSIPSv2 shows the best performance among the three models, and the prediction skill of the correlation is comparable to that of the ANN with the retrospective approach for LD1–LD5. The correlation after LD5 drops quickly for the CanSIPSv2 and is therefore much smaller than that of the ANN. The MAPE of predicted precipitation varies significantly among the three models, ranging from ~ 25% for the CanSIPSv2 to > 100% for the COLA-RSMAS-CCSM4 (Fig. 8b). Systematic errors in the model-simulated precipitation were documented in previous studies (e.g., Zhao et al. 2016, 2017). In addition, the CanSIPSv2 well captures the HSS (> 50%) for LD1–LD7 (Fig. 8c). For anomalous years (precipitation anomalies outside the ± 1 standard deviation), the improvement of the HSS compared to all years is not as much as that in the ANN (Fig. 8d), indicating that the prediction skill for extreme precipitation events in dynamic models is worse than that in the ANN.

Overall, the prediction skill of the ANN model resembles that of the stepwise linear regression model for the lead time within one year, but the former is superior to the latter for some lead times (especially for the leave-three-out approach). The similar prediction skill between the two statistical models indicates a largely linear relationship between UCRB spring precipitation and SST at seasonal and extended seasonal scales. Compared to the NMME models, the ANN shows better or comparable prediction skills for short-lead seasonal prediction (shorter than 6 months), but substantially higher skills for extended seasonal prediction (up to 1 year). The good prediction skills of the ANN at long lead times are probably due to (1) the high correlation between SST and UCRB soil moisture at these lead times and (2) such SST–soil moisture information persisting into the following spring (Fig. 4). The low prediction skills of the NMME models at longer lead times may be associated with initial conditions for these lead times and model drifts (e.g., Hermanson et al. 2018; Manzanas 2020; Ma et al. 2021).

## Concluding remarks

This study investigates the influence of SST over multiple ocean basins on the UCRB spring precipitation and provides an extended seasonal prediction of the precipitation using statistical forecast models. The extratropical North Pacific plays a more significant role in the UCRB spring precipitation than the tropical Pacific and North Atlantic. SST predictors over the three ocean basins are developed and show higher correlations with the UCRB precipitation than the widely used oceanic indices in the literature (e.g., PDO, ONI, and AMO).

Normalized SST predictors of the three basins are applied to the ANN model for predicting the UCRB precipitation using both the leave-three-out and retrospective cross-validations. The ANN model shows good skills for predicting the precipitation up to one year in advance, with correlation > 0.45, MAPE < 20%, and HSS > 50%, respectively. The good prediction skills of the ANN at long lead times may be due to the high correlation between SST and UCRB soil moisture at these lead times and such SST–soil moisture information persisting into the following spring. The ANN shows similar prediction skills to those of the stepwise linear regression model, suggesting a largely linear system between precipitation and SST. The autoregression model shows no skill (correlation < 0.10). The prediction skill by using SST predictors derived in the three ocean basins is superior to the skill by using established modes of variability (i.e., the six oceanic indices). The three NMME models exhibit different skills in predicting the precipitation, but they are all lower than those of the ANN model. The CanSIPSv2 model, the best among the three NMME models, shows moderate skills for the lead time less than eight months, with correlation ~ 0.40, MAPE ~ 25%, and HSS > 40%. The lower prediction skills of the NMME models at longer lead times are probably linked to initial conditions for these lead times and model drifts (e.g., Hermanson et al. 2018; Manzanas 2020; Ma et al. 2021).

In conclusion, the ANN model can predict the UCRB spring precipitation for up to around one year in advance, providing a greater skill than dynamic models. Even though mountain snowpacks are the major source for streamflow (e.g., Barnett et al. 2005), precipitation anomalies over the UCRB during spring are also able to significantly influence the UCRB streamflow during April–July. Currently, the UCRB ensemble streamflow prediction uses historical probabilistic distributions of rainfall and temperature as part of the climate inputs. Improved seasonal prediction of precipitation over the UCRB, such as that presented in this study, could provide more realistic climate inputs to the UCRB streamflow prediction, and so potentially improve its skill, especially for the years with strong precipitation anomalies. Thus, the methods described in this study should improve climate guidance for water resource managers to act within existing fiscal management protocols.

Ongoing and future work include further understanding of the dynamical mechanism behind the statistical relationship between the UCRB precipitation and SSTs. One might need to design experiments such as prescribing SSTs over different basins in GCMs to evaluate the response of the UCRB precipitation to the prescribed SSTs. The future work also includes further improving prediction skills (especially for three- to six-month lead predictions) by applying other linear/nonlinear statistical forecast models (e.g., Ridge and Lasso regressions and convolutional neural network) and incorporating other variables into the model. Moreover, future work may consider a complete investigation of the source of bias and prediction skills of the NMME models.

## Supplementary Information

Below is the link to the electronic supplementary material.

Supplementary file1 (DOCX 3829 kb)

## Data Availability

The NOAA CPC data is from https://psl.noaa.gov/data/gridded/data.unified.daily.conus.html. The NLDAS-2 data is from https://disc.gsfc.nasa.gov/datasets?keywords=nldas&page=1. The UCRB streamflow data comes from https://www.usbr.gov/lc/region/g4000/NaturalFlow/supportNF.html. The HadISST data is from https://www.metoffice.gov.uk/hadobs/hadisst/. The ERA5 data is from https://cds.climate.copernicus.eu/#!/search?text=ERA5&type=dataset. The NMME output is from http://iridl.ldeo.columbia.edu/SOURCES/.Models/.NMME/. The PDO index is from https://www.ncei.noaa.gov/pub/data/cmb/ersst/v5/index/ersst.v5.pdo.dat. The ONI index is from https://origin.cpc.ncep.noaa.gov/products/analysis_monitoring/ensostuff/ONI_v5.php. The AMO index is from https://psl.noaa.gov/data/timeseries/AMO/. The NPI index is from https://climatedataguide.ucar.edu/sites/default/files/npindex_monthly.txt. The NPGO index is from http://www.o3d.org/npgo/npgo.php. The ATL3 index is computed as the domain averaged SST anomaly over the equatorial Atlantic (3°S–3°N, 0°–20°W) using the HadISST data.

## Code availability

For additional questions regarding the code sharing, please contact the corresponding author at siyu_zhao@atmos.ucla.edu.
