# Supplementary material for: Extended seasonal prediction of spring precipitation over the Upper Colorado River Basin
Source: Clim Dyn. 2022 Jul 21;60(5-6):1815–29. doi: 10.1007/s00382-022-06422-x (PMC10011310; doi:10.1007/s00382-022-06422-x)
Supplement: Supplementary file 1 — Supplementary file1 (DOCX 3829 kb) [file 382_2022_6422_MOESM1_ESM.docx]

**Supplementary material**

**Extended Seasonal Prediction of Spring Precipitation over the Upper Colorado River Basin**

**Siyu Zhao^1^, Rong Fu^1^, Michael L. Anderson^2^, Sudip Chakraborty^3^, Jonathan H. Jiang^3^, Hui Su^3^, and Yu Gu^1^**

^1^Department of Atmospheric and Oceanic Sciences, University of California, Los Angeles, Los Angeles, CA, USA

^2^California Department of Water Resources, Sacramento, CA, USA

^3^Jet Propulsion Laboratory, California Institute of Technology, Pasadena, CA, USA

* Siyu Zhao

siyu_zhao@atmos.ucla.edu


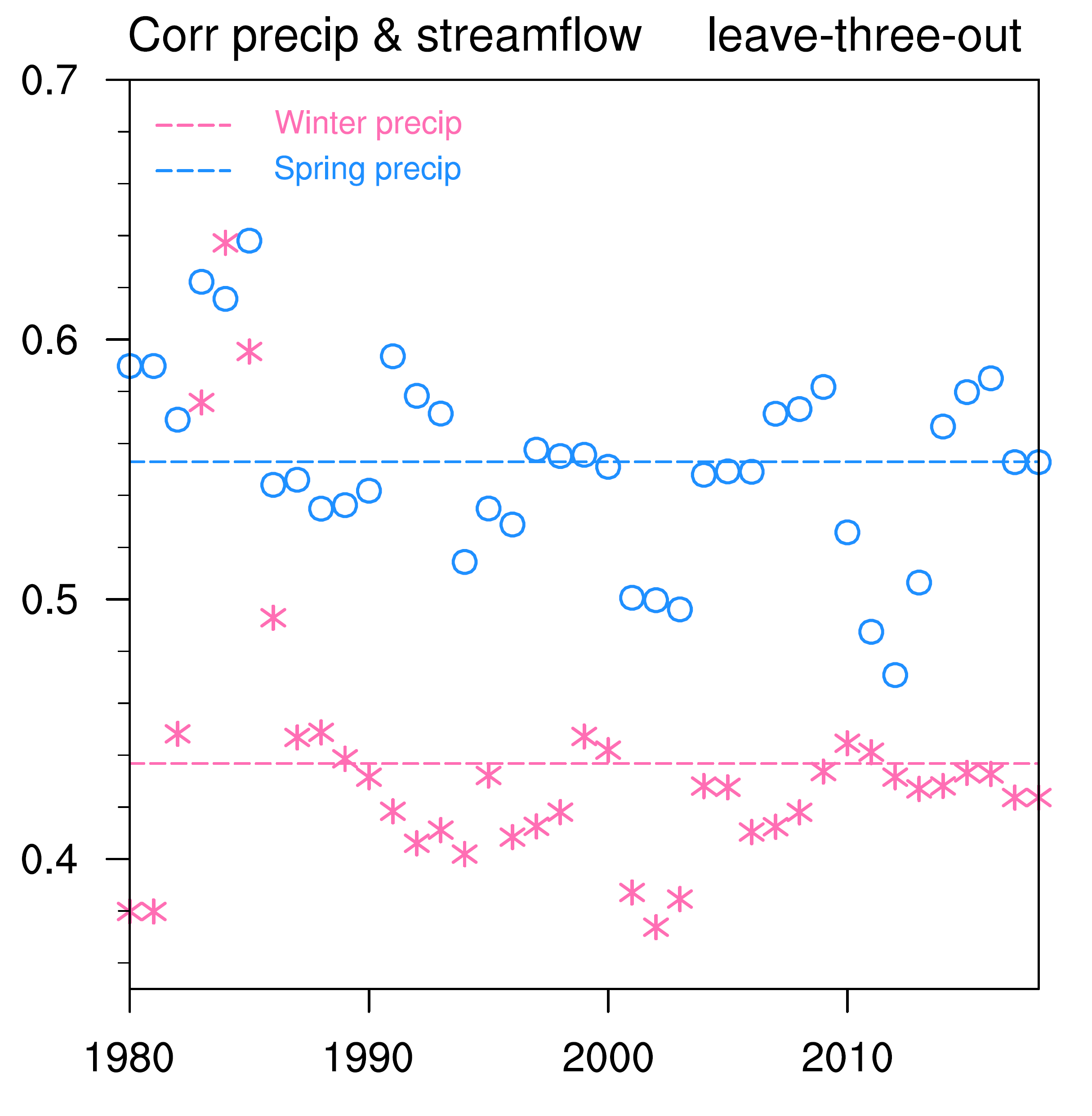


**Fig. S1** Correlation coefficients between the UCRB averaged precipitation during winter (pink) and spring (blue), respectively, and April–July normalized total natural flow at Lees Ferry using the leave-three-out cross-validation.


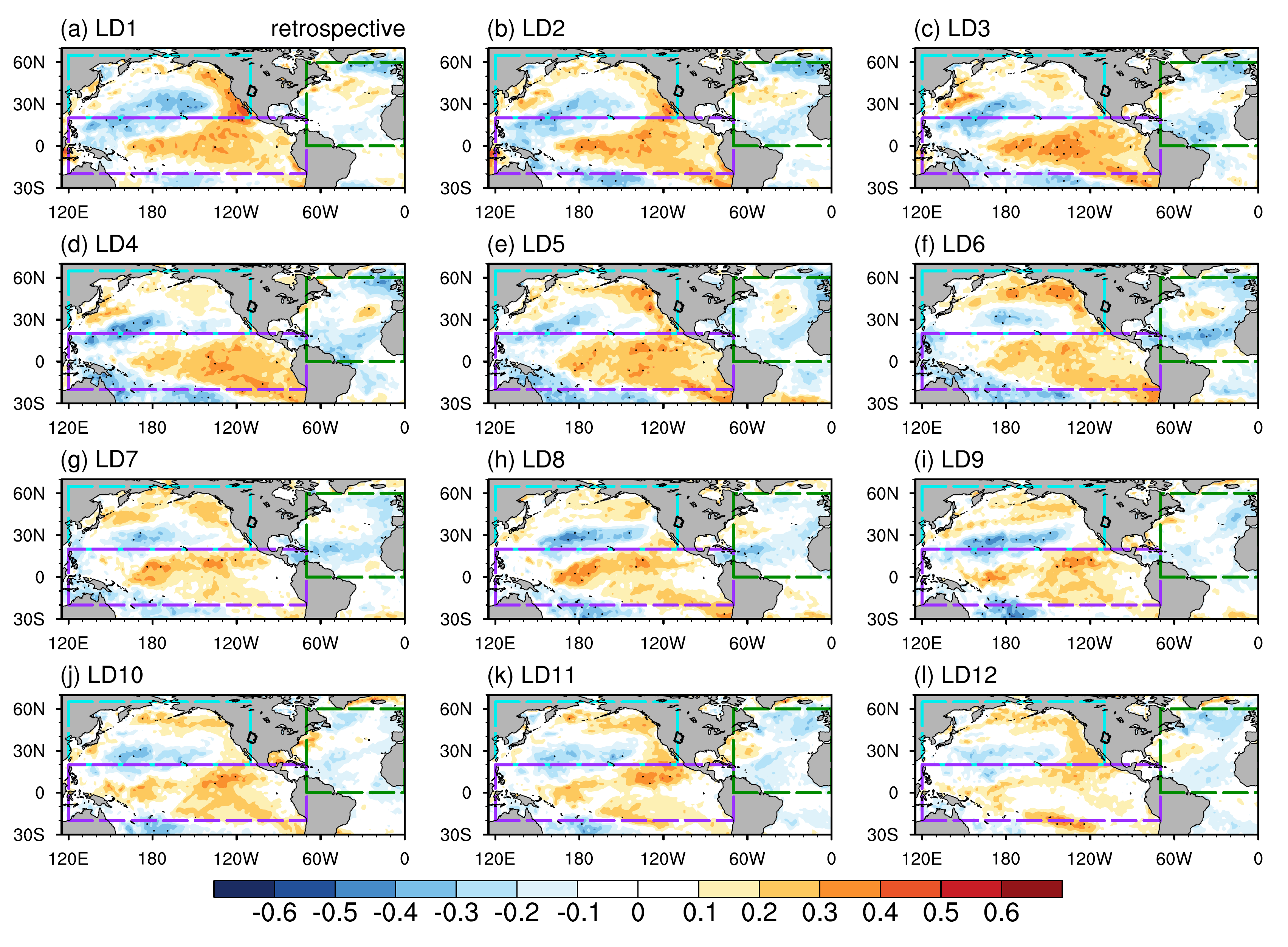


**Fig. S2 a** The correlation coefficient (shading) between the UCRB spring precipitation and SST at LD1 using the retrospective approach averaged for the period of 1980–2019. Black dots denote correlation coefficients significant at the 0.05 level. The blue, purple, and green boxes represent the extratropical North Pacific, tropical Pacific, and North Atlantic, respectively. **b–l** As in **a**, but from LD2 to LD12.


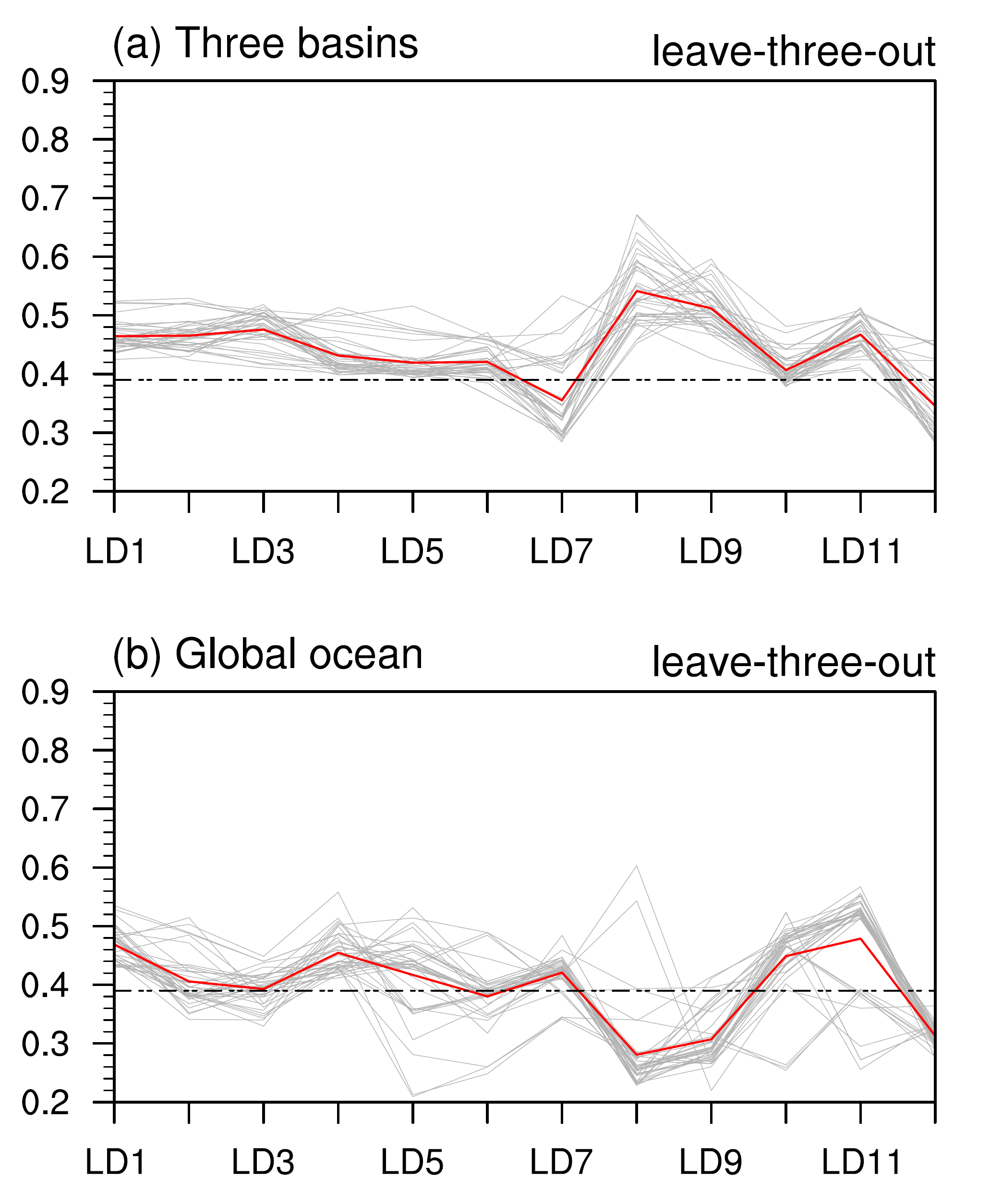


**Fig. S3 a** Correlation coefficients between the SST predictors over the three basins and UCRB averaged spring precipitation from LD1 to LD12 for 1980–2019. The gray lines denote correlations between the two variables using the leave-three-out cross-validation. There are a total of 40 gray lines and each line represents one year during 1980–2019. The red line represents the average of all the 40 years. **b**, Same as in **a**, but for the SST predictors over global oceans.


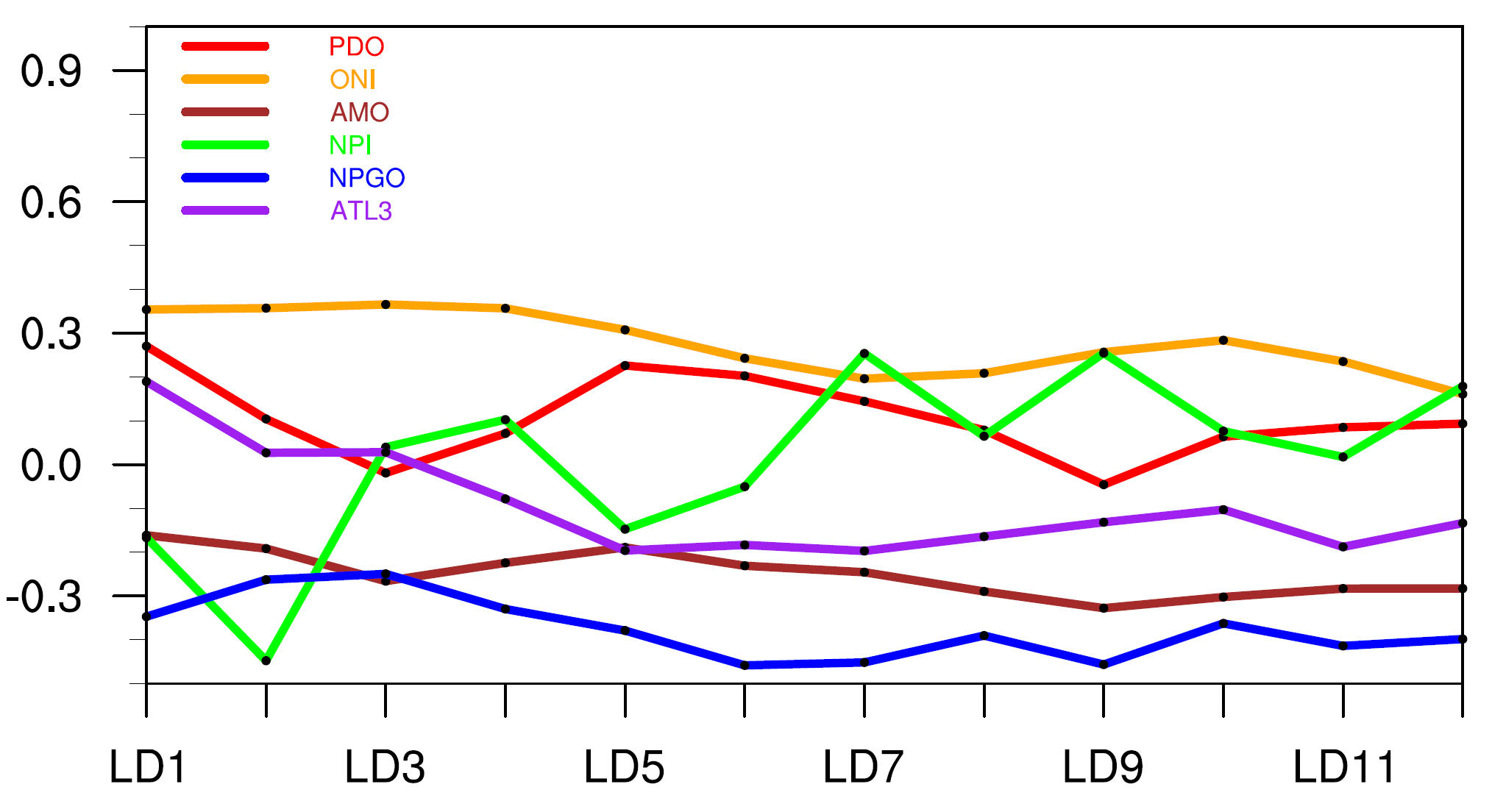


**Fig. S4** Correlation coefficients between six oceanic indices and UCRB averaged spring precipitation from LD1 to LD12 for 1980–2019.


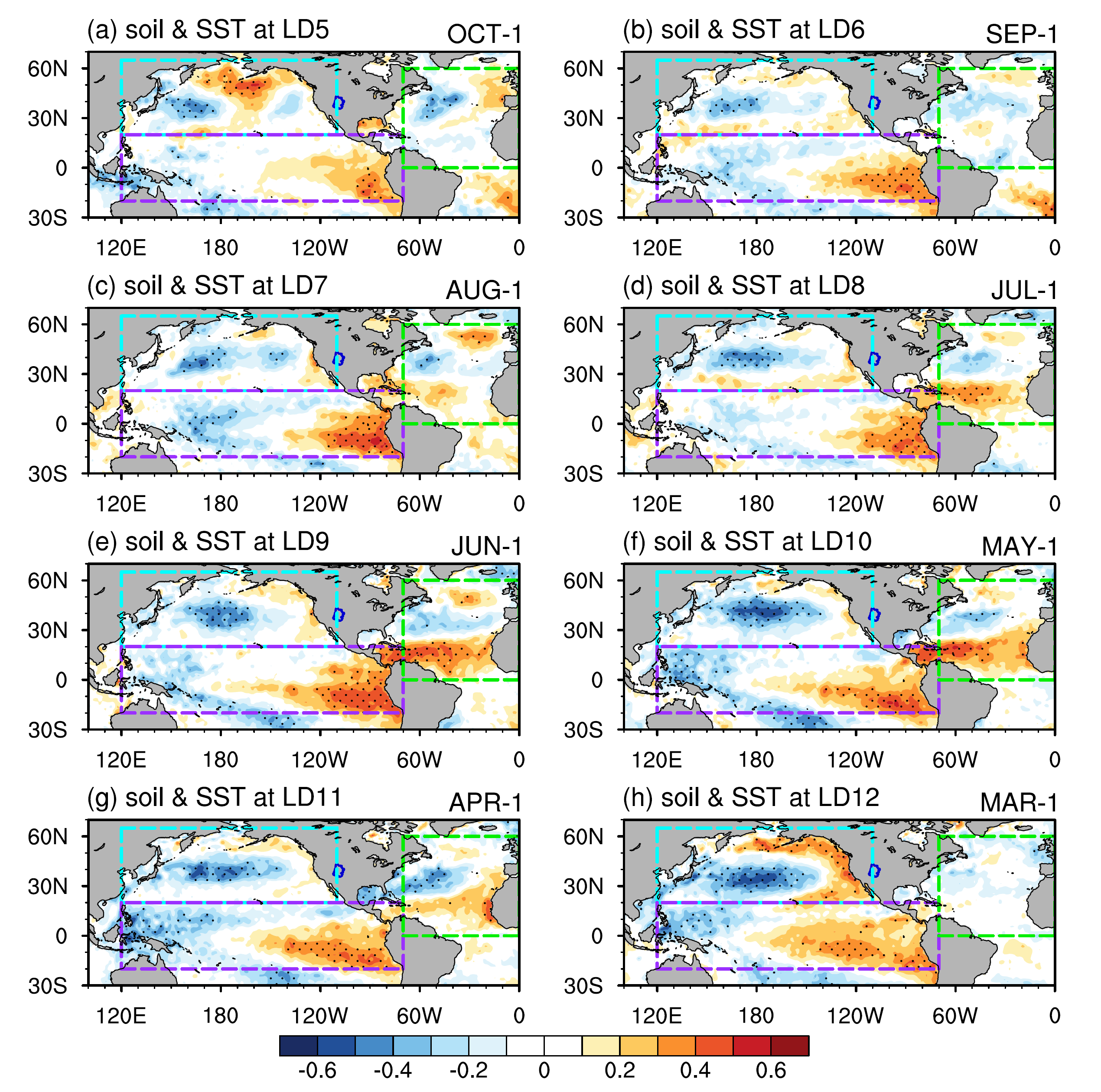


**Fig S5 a** The correlation coefficient (shading) between SST and UCRB averaged soil moisture at LD5 for the period of 1980–2019. Black dots represent correlation coefficients significant at the 95% level. The blue, purple, and green boxes represent the extratropical North Pacific, tropical Pacific, and North Atlantic, respectively. **b–h** Same as in **a**, but for LD6 to LD12.


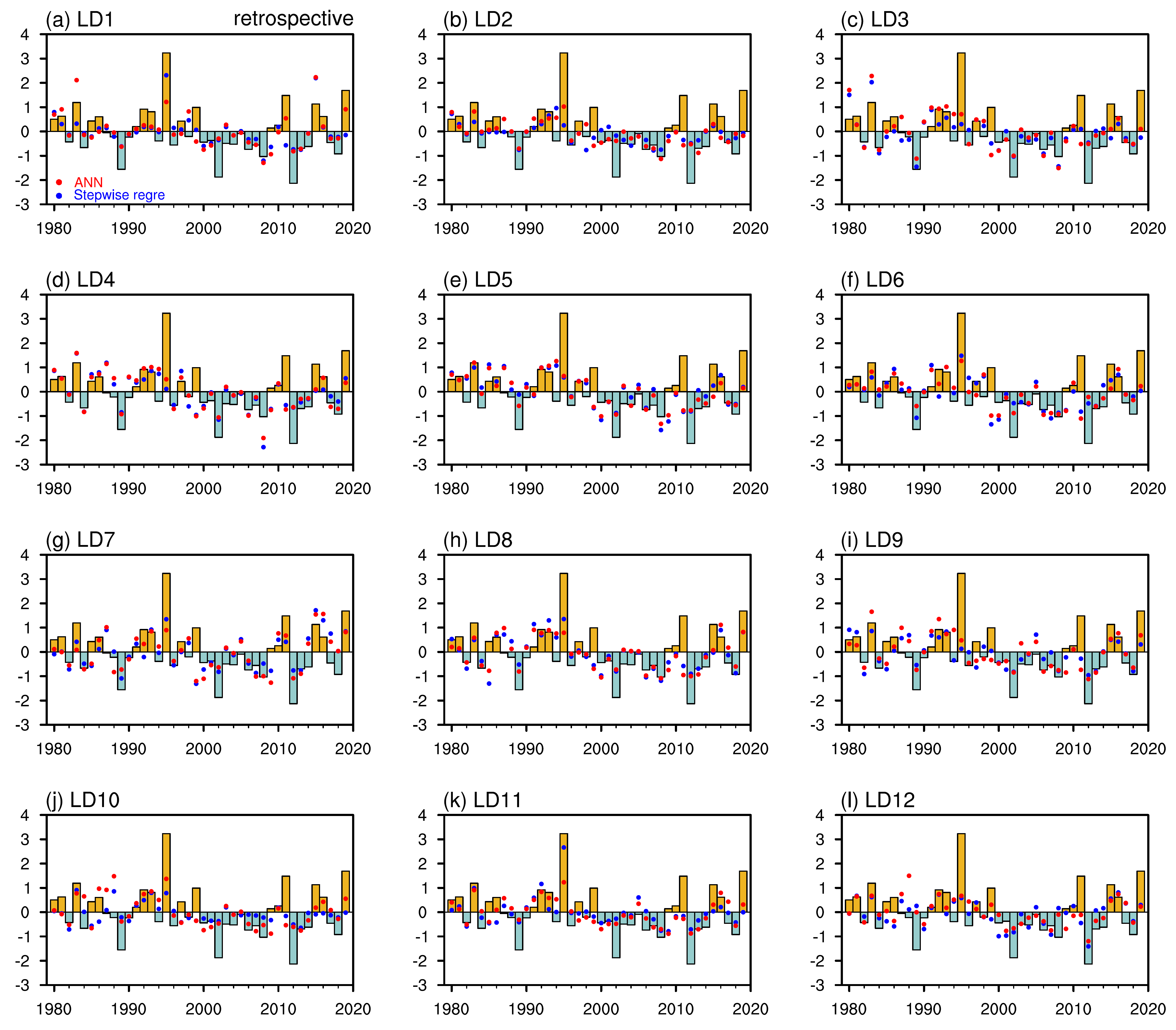


**Fig S6 a** The observed normalized UCRB spring precipitation (bars) and predicted results by the ANN (red dots) and stepwise linear regression (blue dots) by using SST predictors for the three ocean basins at LD1 from 1980 to 2019 with the retrospective cross-validation. **b–l** As in **a**, but from LD2 to LD12.

| Lead | LD1 | LD2 | LD3 | LD4 | LD5 | LD6 | LD7 | LD8 | LD9 | LD10 | LD11 | LD12 |
| --- | --- | --- | --- | --- | --- | --- | --- | --- | --- | --- | --- | --- |
| Correlation | 0.25 | 0.23 | 0.19 | 0.22 | 0.20 | 0.24 | 0.20 | 0.15 | 0.19 | 0.18 | 0.20 | 0.19 |
| MAPE % | 20.5 | 20.8 | 21.2 | 20.8 | 20.7 | 20.6 | 21.0 | 21.8 | 21.5 | 21.5 | 20.8 | 21.0 |
| HSS % | 41.5 | 41.0 | 33.3 | 38.5 | 33.6 | 39.8 | 34.1 | 26.9 | 32.8 | 27.7 | 34.0 | 33.0 |
| HSS (>1std years) % | 42.0 | 39.5 | 32.5 | 37.9 | 33.4 | 39.3 | 33.0 | 25.1 | 33.2 | 28.2 | 34.7 | 23.1 |

**Table S1** The correlation coefficients, MAPE (%), HSS (%) for all years, and HSS (%) for anomalous years between the observed and predicted precipitation from LD1 to LD12 using six oceanic indices (PDO, ONI, AMO, NPI, NPGO, and ATL3) obtained from the ANN (10 ensemble mean). The leave-three-out cross-validation approach is used.
